# An End-to-End General Language Model (GLM)-4-Based Milling Cutter Fault Diagnosis Framework for Intelligent Manufacturing

**DOI:** 10.3390/s25072295

**Published:** 2025-04-04

**Authors:** Jigang He, Xuan Liu, Yuncong Lei, Ao Cao, Jie Xiong

**Affiliations:** 1 School of Management Science and Engineering, Southwestern University of Finance and Economics, Chengdu 611130, China; xiongjie@swufe.edu.cn; 2 Institute of Chinese Financial Studies, Southwestern University of Finance and Economics, Chengdu 611130, China; 3 School of Mechanical Engineering, Southwest Jiaotong University, Chengdu 610031, China; yuncong_lei@my.swjtu.edu.cn (Y.L.); ao@my.swjtu.edu.cn (A.C.)

**Keywords:** large language models (LLMs), cutting life default diagnosis, multi-feature fusion

## Abstract

CNC machine and cutting tools are an indispensable part of the cutting process. Their life default diagnosis is related to the efficiency of the entire production process, which ultimately impacts economic performance. Many methods provided by deep learning articles have been verified for use on large cutting datasets and can help in diagnosing tools’ lifetime well; however, on small samples, the challenge of learning difficulties still emerges. The rise in large language models (LLMs) has brought changes to tool life diagnosis. This study proposes a fault diagnosis algorithm based on GLM-4, and the experimental validation on the PHM 2010 dataset and a proprietary milling cutter dataset demonstrates the superiority of the proposed model, achieving diagnostic accuracies of 93.8% and 93.3%, respectively, outperforming traditional models (SVM, CNN, RNN) and baseline LLMs (ChatGLM2-6B variants). Further robustness and noise-resistance analyses confirm its stability under varying SNR levels (10 dB to −10 dB) and limited training samples. This work highlights the potential of integrating domain-specific feature engineering with LLMs to advance intelligent manufacturing diagnostics.

## 1. Introduction

Tool processing is an essential aspect of daily production scenarios, and accurately diagnosing tool life failures enables processing factories to make timely and informed decisions. With the rise of artificial intelligence technology, deep learning has demonstrated significant advancements in the machinery sector. These techniques possess remarkable fitting capabilities for handling complex, large, and multi-signal data, effectively extracting features hidden within the signals to provide a reliable basis for monitoring and predicting tool life accurately [1]. First, deep learning can be combined with traditional mechanical classification methods or monitoring methods to help accurately determine the tool wear stage and classification [2,3,4]. Not only that, but deep learning can also be used to optimize cutting parameters [5], better handle noise [6], better fit tool wear values [7], and interact with the processing environment. Among these, the most intuitive use is the accurate monitoring and prediction of wear [8,9]. Some methods use deep learning combined with sensors to apply the surface roughness of the tool to judge the wear [10]; some use integrated models to fuse features from multiple sensors [11] and enhance the generalization of tool wear prediction through deep learning algorithms [12]; and some use deep learning of multi-channel signals to convert time domain signals into two-dimensional time-frequency domain signals, perform splicing training with the number of cutting times after the dimensionality increase, and obtain the effect of significantly reducing the prediction time through the loss function [13]. The above methods are just the tip of the iceberg of how deep learning can help improve the monitoring and prediction of tool wear. Facts have proven that the characteristics of deep learning such as automatic feature extraction, multi-source data fusion, and dynamic adaptability can indeed help to effectively monitor and predict tool wear when there are sufficient cutting processing data.

However, this robust functionality encounters challenges in the context of small sample sizes, particularly with unbalanced samples [14]. The conditions of each tool processing operation can vary significantly, influenced by factors such as temperature, noise, workpiece surface conditions, vibration, and so on. Some methods, such as the multi-dimensional hybrid intelligent diagnosis method [15], Time-Spectrum Domain Adaptation Network (TSDAN) [16], transformer network and auxiliary classifier generative adversarial network (TRA-ACGAN) [17], and hybrid data augmentation mechanism (HDAM) [18], have contributed to solving the challenges of small samples and cross-conditions and have improved the adaptability of the model to a certain extent. Nonetheless, they are generally ineffective when facing extreme situations, and in small sample scenarios, excessive variations in a single factor can disproportionately impact the model, leading to reduced fitting accuracy and complicating fault diagnosis.

In addition, another challenge of small samples is that the proposed models and methods have poor migration capabilities when facing non-specific datasets, or in other words, across datasets, which in turn affects their generalization. Many transfer models, such as deep convolutional transfer learning network (DCTLN) [19] and joint distribution adaptation-based transfer network with diverse feature aggregation (JDFA) [20], and adversarial models, such as Dynamic Multi-Adversarial Adaptation Network (DMAAN) [21] and deep adversarial subdomain adaptation network (DASAN) [22], attempt to solve the problem of cross-data. Consequently, achieving high-precision diagnostics in the face of limited fault samples continues to be a critical focus for the industry.

**Figure 1:**
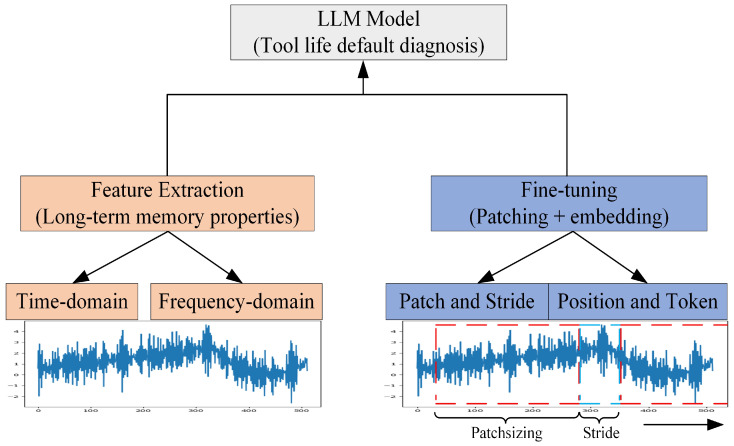
Model framework.

Recently, as the advantages of large language models (LLMs) in processing sequence data have been continuously proven [23,24], large language models have begun to be put into practice in the mechanical field [25,26]. This study proposes a fault diagnosis algorithm based on GLM-4, as shown in Figure 1.

Recently, the advent of large models has marked a paradigm shift in milling cutter fault diagnosis. Traditional deep learning methods, such as CNNs and RNNs, have demonstrated effectiveness in extracting complex features and modeling temporal dependencies. However, the scale and pre-trained nature of large language models (LLMs) have opened new avenues for enhancing diagnostic performance. By leveraging massive amounts of pre-learned contextual knowledge and advanced attention mechanisms, large models not only capture global patterns across multi-sensor data but also provide robust semantic understanding even under small sample conditions. This trend is exemplified by recent studies that apply LLMs to industrial fault diagnosis tasks, where they achieve superior performance in handling noise, variability, and cross-domain generalization compared to conventional architectures. Thus, integrating large model technology into milling cutter fault diagnosis represents an exciting development that bridges state-of-the-art natural language processing techniques with practical manufacturing applications.

This study presents a novel framework for milling cutter fault diagnosis, addressing critical challenges in small-sample industrial scenarios. The key contributions are threefold:

**Semantic Transformation of Numerical Signals:** The GLM-4 Feature Extraction Module (GLM-4-FE) transforms raw vibration signals into semantically rich linguistic descriptions, bridging traditional numerical diagnostics with advanced language models. This transformation enhances interpretability, preserves feature significance, and improves classification accuracy and adaptability across diverse fault conditions.

**Adaptive Multi-Domain Feature Fusion:** The framework’s adaptive feature fusion mechanism (FE) integrates time-domain, frequency-domain, and time–frequency-domain features, capturing both short-term bursts and long-range temporal dependencies. This comprehensive fusion provides robust representations capable of detecting complex failure modes, enhancing the predictive accuracy and versatility in dynamic industrial settings.

**Time-Series Fine-Tuning for Robustness:** By employing time-series fine-tuning (TS), the framework refines its sensitivity to temporal variations, detecting abrupt changes and non-stationary signals with precision. Combined with multi-head attention in pre-trained models, this approach ensures robustness against noise, strong adaptability to transfer learning, and stable diagnostic performance even in data-scarce or noisy environments.

The remainder of this paper is structured as follows: Section 2 details the architecture of the proposed fault diagnosis framework and its key components. Section 3 covers the experimental setup, including the datasets (PHM2010 and milling cutter data) and baseline models (SVM, CNN, RNN, ChatGLM2), followed by a comprehensive evaluation comparing the proposed model’s accuracy, robustness, and generalization across various conditions. Section 4 concludes by summarizing the key contributions and potential applications in industrial fault monitoring and predictive maintenance.

## 2. Model Construction

### 2.1. Framework of Feature-Based GLM-4

#### 2.1.1. Feature Extraction

Unlike the traditional GLM-4 framework that primarily handles semantic text, GLM-4-FE leverages time-domain vibration signals collected from bearings, represented as discrete data points by sensors. To fully exploit GLM-4’s capability in processing semantic information, we extracted interpretable feature information from the raw vibration signals. Regarding feature selection, we focused on time-domain, frequency-domain, and time–frequency-domain features, as shown in Table 1. This selection is based on two main reasons: firstly, the extraction of complex features is relatively cumbersome and may require tailored designs specific to the characteristics of the vibration data; secondly, we aim to fully utilize GLM-4’s robust learning, extraction, and classification capabilities by employing easily obtainable time-domain and frequency-domain features for effective fault diagnosis. Time-domain features reflect the instantaneous variations of the signal, while frequency-domain features reveal the periodicity and frequency components, providing a more comprehensive description of the fault signal characteristics when combined.

#### 2.1.2. Semantic Description

After extracting features, we proceeded with the dataset construction and fine-tuning steps to convert these numerical features into a format that GLM-4 can comprehend using linguistic descriptions instead of raw numerical values. We combined the extracted time–frequency-domain feature values with their corresponding textual descriptions and used the fault mode as labels for supervised learning, forming question–answer pairs as inputs. This approach not only preserves the physical significance of the features, avoiding information loss typically associated with feature standardization, but also enables GLM-4 to more accurately understand and process input data in new tasks. To minimize the model’s reliance on expert knowledge, the textual descriptions serve merely as illustrative examples, primarily aimed at helping the LLM comprehend the task requirements and the meaning of the input data. Through this transformation from numerical features to linguistic descriptions, GLM-4-FE’s strengths in language understanding and semantic reasoning can be effectively leveraged, achieving efficient industrial fault diagnosis.

In conclusion, GLM-4-FE facilitates a seamless transition from raw vibration data to a semantically rich representation by integrating feature extraction and linguistic fine-tuning strategies. This not only enhances the interpretability of the features but also fully utilizes GLM-4’s advanced capabilities in learning and classification, making it a powerful tool for fault diagnosis in complex industrial environments. Future work may involve further optimizing the feature extraction methods and fine-tuning strategies to enhance the model’s diagnostic accuracy and adaptability, thereby meeting the increasingly diverse and complex requirements of industrial applications.

### 2.2. Framework of Data-Based GLM-4

#### 2.2.1. Patching

Industrial vibration signals often exhibit high sampling frequencies and large data sizes, which can introduce substantial redundancy and high computational costs when fed directly into the model. To address this, adjacent points are grouped into patches to capture local features and reduce the overall sequence length.

Let $X\in R^{T}$ denote the preprocessed vibration signal. Define the patch size as P, and split X into patches of length P:

(1) $${Patch}_{i}=\left[x_{\left(i-1\right)P+1}^{\prime },x_{\left(i-1\right)P+2}^{\prime },\ldots ,x_{iP}^{\prime }\right],i=1,2,\ldots ,N$$

where N=T/P. If the last segment contains fewer than P points, it is truncated. Subsequently, a one-dimensional convolution can be applied to extract feature vectors from each patch directly:

(2) $$z_{i}=f_{\theta }\left({Patch}_{i}\right),z_{i}\in R^{d_{patch}}$$

where $f_{\theta }$ represents a one-dimensional convolution or any suitable neural network, and $z_{i}$ is the feature representation of the i-th patch.

#### 2.2.2. Token Embedding

To enable the GLM-4 Transformer backbone to process these numerical features, each $z_{i}$ must be projected to the model’s input dimension $d_{model}$. A linear mapping is commonly used:

(3) $$e_{i}=W_{E}z_{i}+b_{E}$$

where $W_{E}\in R^{d_{model}\times d_{patch}}$ and $b_{E}\in R^{d_{model}}$. If the patching procedure already produces $z_{i}$ with dimensionality $d_{model}$, this linear layer can be skipped. The resulting sequence of embedded patch vectors is as follows:

(4) $$E={\left[e_{1},e_{2},\ldots ,e_{N}\right]}^{T}\in R^{{N\times d}_{patch}}$$

#### 2.2.3. Positional Embedding

Because the Transformer architecture itself does not inherently encode the ordering of input tokens, it is necessary to inject positional information explicitly. For time-series data, a sinusoidal positional embedding is often employed, embedding the position directly into the sequence representation. For the i-th patch (indexing from 1 or 0), the position encoding can be defined as follows:

(5) $$\left\{\begin{array}{c} p_{i.2k}=sin\left({i/10000}^{2k/d_{model}}\right),\\ p_{i.2k+1}=cos\left({i/10000}^{2k/d_{model}}\right), \end{array}\right.k=0,\ldots ,\frac{d_{model}}{2}-1$$

Writing this in vector form yields $p_{i}\in R^{d_{model}}$. The final input to the Transformer’s first layer is obtained by adding each patch embedding $e_{i}$ to its positional vector:

(6) $$h_{i}^{\left(0\right)}=e_{i}+p_{i},i=1,\ldots ,N$$

#### 2.2.4. Transformer Blocks

Consider a multi-layer Transformer with L layers. From layer 1 to L, each layer’s computation can be summarized as follows. Let $H^{\left(l-1\right)}\in R^{{N\times d}_{model}}$ denote the output of the (l−1)-th layer.

**Multi-head attention**

(7) $${Q=H}^{\left(l-1\right)}W_{Q},K=H^{\left(l-1\right)}W_{K},V=H^{\left(l-1\right)}W_{V}$$

where $W_{Q},W_{K},W_{V}\in R^{d_{model}\times d_{model}}$. The attention mechanism is given by the following:

(8) $$Att(Q,K,V)=softmax(\frac{{QK}^{T}}{\sqrt{d_{k}}})V$$

where $d_{k}$ is the dimensionality per attention head. If there are h heads, their outputs are concatenated and then linearly projected to produce the final attention output M.

**Residual Connection and Feed-Forward Network (FFN)**

(9) $${H^{\prime \left(l\right)}=H}^{\left(l-1\right)}+M$$

(10) $${H^{\left(l\right)}=H}^{\prime \left(l\right)}+FFN(H^{\prime (l)})$$

Here, the FFN typically consists of two linear layers with an activation function in between.

This process is repeated through layers 1 to L, finally yielding the following:

(11) $$H^{\left(L\right)}={\left[H_{1}^{\left(L\right)},H_{2}^{\left(L\right)},\ldots ,H_{N}^{\left(L\right)}\right]}^{T}$$

which serves as the Transformer’s top-layer output.

#### 2.2.5. Classification Head

For fault diagnosis, the model must predict a discrete fault category. A classification head can be added on top of $H^{\left(L\right)}$. Average or max-pool all patch outputs to form a global representation:

(12) $$h_{pool}=\frac{1}{N}\sum_{i=1}^{N}H_{i}^{\left(L\right)}$$

which is then fed into a classification layer. Introduce a learnable token at the start of the sequence. After propagation through the Transformer, $H_{CLS}^{\left(L\right)}$ is taken as the global feature vector. Let $h_{agg}\in R^{d_{model}}$ denote the aggregated representation. A linear mapping followed by softmax is applied:

(13) $$\hat{y}=softmax\left({W_{c}h}_{pool}+b_{C}\right),W_{c}\in R^{{C\times d}_{model}},b_{C}\in R^{C}$$

where C denotes the number of fault categories.

#### 2.2.6. Training and Inference

Suppose the training set is ${\left\{\left(X^{\left(m\right)}{,y}^{\left(m\right)}\right)\right\}}_{m=1}^{M},where\;y^{\left(m\right)}\in \left\{1,\ldots ,C\right\}$ indicates the ground-truth fault category. A standard cross-entropy loss is employed:

(14) $$L=-\sum_{m=1}^{M}\sum_{c=1}^{C}H\left[y^{\left(m\right)}=c\right]ln\left({\hat{y}}_{c}^{\left(m\right)}\right)$$

in which ${\hat{y}}_{c}^{\left(m\right)}$ is the predicted probability for category c on sample m, and H is the indicator function. Given the vast number of parameters in GLM-4, low-rank adaptation (LoRA) can be applied to reduce memory and computational overhead. Only a subset of attention projection or feed-forward parameters is trained, while the majority of model weights remain frozen-beneficial for large-scale industrial fault diagnosis scenarios.

Overall, this workflow—encompassing patching, embedding, positional encoding, and a Transformer-based backbone—enables efficient and accurate fault diagnosis by leveraging the expressive power of large-scale language models while addressing the unique challenges of high-frequency industrial vibration data.

## 3. Experiment and Results

### 3.1. Case 1: PHM 2010

#### 3.1.1. Dataset Description

For validation purposes, the performance of the proposed model is first tested using the PHM 2010 public dataset [27], where the experimental parameters are shown in Table 2, and the cutting conditions of the dataset remain unchanged.

#### 3.1.2. Comprehensive Evaluation

**Figure 2:**
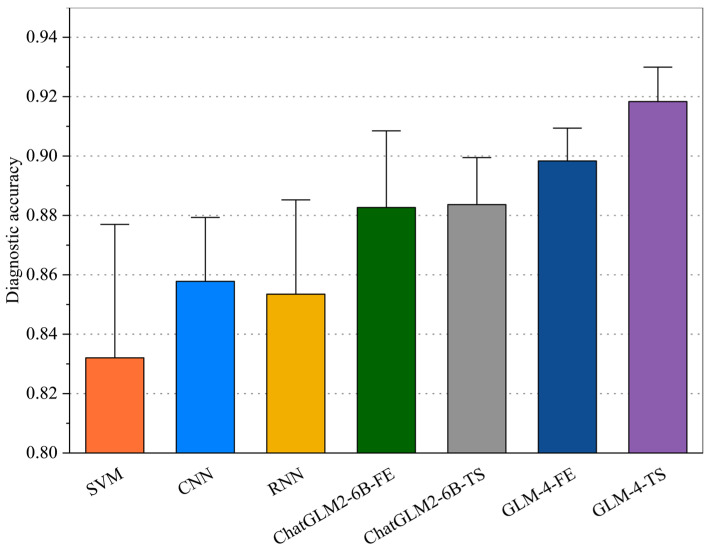
Diagnostic accuracy of each model in Case 1.

The diagnostic accuracy of each model in Case 1 is shown in Figure 2 and Table 3. In experiments on the PHM2010 dataset, SVM, CNN, and RNN represent more traditional machine learning and early deep learning approaches. Their accuracies are relatively stable but not outstanding, with SVM mostly fluctuating between 0.79 and 0.87, CNN showing a slight improvement, and RNN benefiting from temporal dependencies yet still limited in its feature extraction capabilities. As large models such as ChatGLM2-6B and GLM-4 enter the comparison, there is a marked increase in accuracy. In particular, ChatGLM2-6B-TS and GLM-4-FE exhibit strong adaptability in handling temporal information and feature representation, typically achieving scores above 0.88–0.90. Notably, when advanced feature extraction and time-series fine-tuning strategies are integrated, GLM-4 and its variants consistently reach 0.92–0.93, demonstrating their potential in multi-scale temporal capture and feature fusion.

From a deeper perspective, large models leverage multi-head attention mechanisms and extensive pretrained parameters to effectively capture multi-dimensional correlations in fault signals across different time spans, covering both short-term fine-grained features and long-range dependencies. At the same time, GLM-4-FE further reduces redundancy in raw signals, enhancing discriminative power across various fault modes; optimizing for GLM-4-TS strengthens the model’s ability to detect non-stationarity and abrupt changes. These factors collectively give the GLM-4 series a strong advantage in robustness and predictive accuracy, making them well suited to the stringent demands of industrial fault diagnosis.

### 3.2. Case 2: Milling Cutter Experiment

#### 3.2.1. Dataset Description

A high-quality cutter’s lifetime cycle can generate a small error (ε) in the economic output of the subsequent time series. The experimental data source of the milling cutter used in this study comes from real processing data. The milling cutter under consideration is the APMT1135 carbide cutter, a product of Duracarb. Its fundamental parameters include a tool tip angle of 85 degrees, a blade relief angle of 11 degrees, a blade length of 11 mm, a thickness of 3.5 mm, an inscribed circle diameter of 6.35 mm, and a maximum cutting depth of 9 mm.

#### 3.2.2. Comprehensive Evaluation

**Figure 3:**
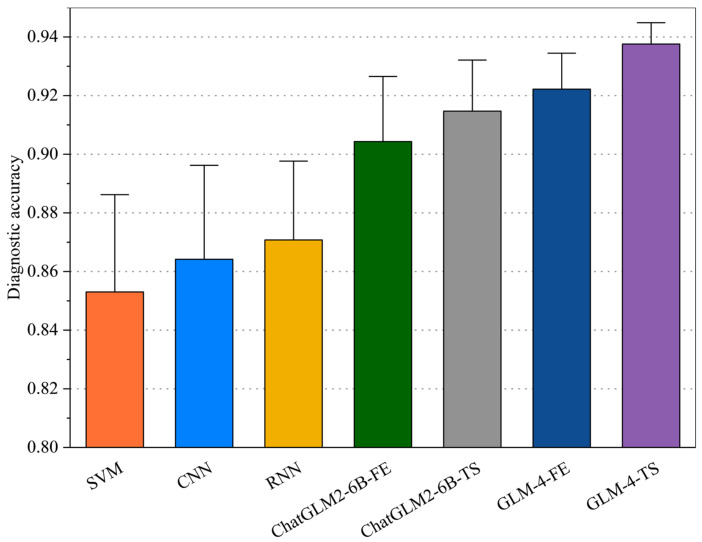
Diagnostic accuracy of each model in Case 2.

The diagnostic accuracy of each model in Case 2 is shown in Figure 3 and Table 4. In this experiment, SVM, CNN, and RNN—representing traditional and early deep learning approaches—exhibit relatively stable yet limited performance. In contrast, ChatGLM2-6B-FE and ChatGLM2-6B-TS more effectively capture multi-dimensional features in fault signals, typically reaching accuracies around 0.90. Notably, ChatGLM2-6B-TS integrates refined TS fine-tuning, enhancing the model’s ability to detect subtle temporal variations. Meanwhile, GLM-4-FE demonstrates superior generalization in multi-scale FE, balancing both local bursts and overall trends.

Building upon this foundation, GLM-4-TS leverages the global attention of large pre-trained models and time-domain adaptation in unison, pushing the average accuracy beyond 0.93. The underlying reasons are twofold: on the one hand, multi-head attention uncovers intricate dependencies in fault data; on the other hand, the synergy of FE and TS fine-tuning reduces noise and redundancy, allowing flexible transitions between short- and long-term features. This multi-module strategy grants GLM-4-TS heightened robustness and sensitivity to varied failure patterns, offering a highly promising solution for industrial applications.

### 3.3. Performance Analysis

#### 3.3.1. Robustness Analysis

**Figure 4:**
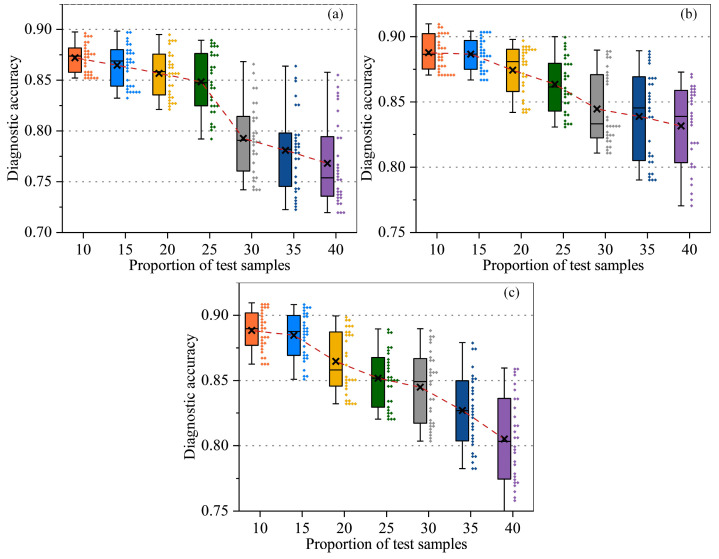
Diagnosis accuracy of deep learning models in different sample proportions (**a**–**c**).

In Figure 4, we compare SVM, CNN, and RNN at various test set proportions (from 10% to 40%) to assess each model’s robustness and adaptability under different data splits. Overall, as the test set ratio increases and the training set shrinks, the average accuracy tends to decrease. SVM exhibits relatively large fluctuations—sometimes maintaining around 0.88–0.89 accuracy at lower test ratios but becoming prone to overfitting or underfitting when the data are limited. CNN shows certain advantages in capturing local features, especially under 15% or 20% test splits, thanks to its convolutional structure for extracting signal patterns. RNN, leveraging temporal dependency modeling, achieves higher accuracy in certain splits but experiences performance swings when the training data become insufficient.

**Figure 5:**
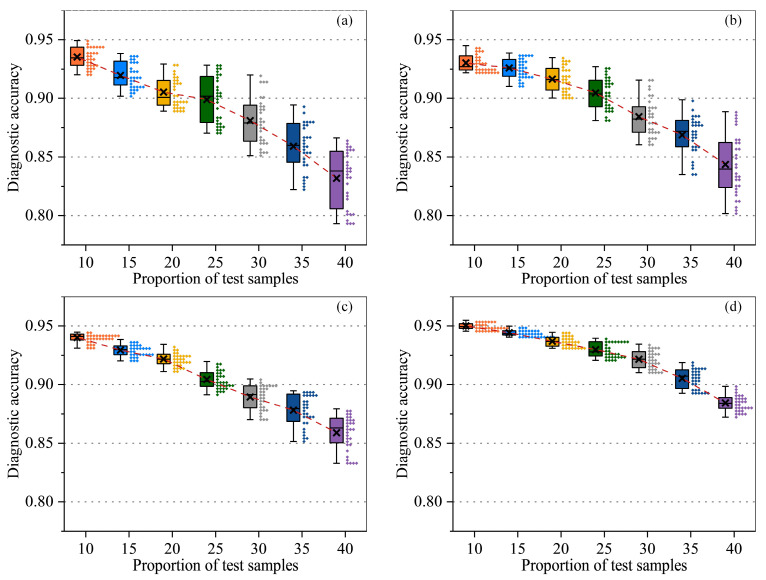
The diagnostic accuracy of LLMs under different sample proportions (**a**–**d**).

In Figure 5, we examined ChatGLM2-6B-FE, ChatGLM2-6B-TS, GLM-4-FE, and GLM-4-TS under various test set ratios to evaluate their fault diagnosis performance. Overall, higher accuracies are observed when the training set is larger (i.e., a smaller test ratio). Models that incorporate FE tend to exhibit stable performance across diverse signal scenarios, while those applying TS fine-tuning excel at capturing temporal dependencies and short-term fluctuations, enhancing the detection of impulsive faults and nonstationary patterns. As the test proportion increases, the available training data decrease, generally leading to a drop in accuracy; however, performance differences among the models become more pronounced.

Notably, GLM-4-TS consistently maintains average accuracies above 0.93 across most test ratios, surpassing ChatGLM2-6B-TS and GLM-4-FE. This advantage likely stems from the synergy between the large-scale pretrained model’s ability to capture global context via multi-head attention and the TS-driven refinement of time-domain features. Specifically, the former uncovers multi-scale dependencies in fault signals, while the latter focuses on local dynamics and temporal evolution. Even at a 40% test ratio, when all models face a significant accuracy drop, GLM-4-TS demonstrates superior robustness through integrated adaptations and deeper feature representation, making it well suited for industrial requirements that prioritize both limited data and high diagnostic precision.

#### 3.3.2. Noise Resistance Analysis

**Figure 6:**
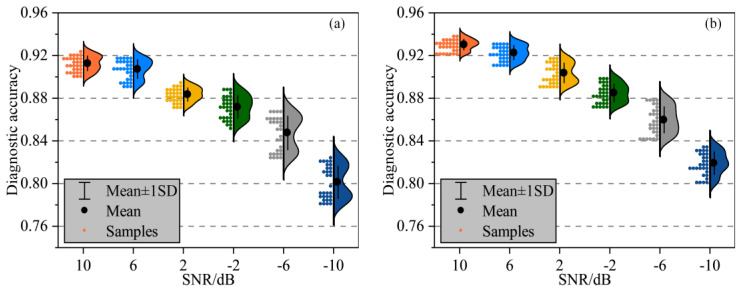
Diagnostic accuracy of GLM-4-TS under different SNRs (**a**,**b**).

In Figure 6, we compare GLM-4-FE and GLM-4-TS under varying signal-to-noise ratios (SNR ranging from 10 dB down to −10 dB). As noise levels increase and the SNR decreases, the overall accuracy declines: while both models can maintain around 0.90 accuracy at a higher SNR, the performance tends to drop significantly in extremely noisy conditions such as −10 dB. Nonetheless, GLM-4-TS demonstrates greater robustness in most noisy scenarios, indicating that TS fine-tuning enhances the model’s sensitivity to subtle signal distortions and random interference.

The superior performance of our approach can be attributed to the synergistic integration of FE and TS. Specifically, GLM-4-TS exploits the large model’s multi-head attention to capture global contextual patterns, while its TS component effectively adapts to local signal variations and nonstationary behaviors. In low SNR conditions—where critical information is increasingly masked by noise—traditional features alone struggle to maintain discriminative power. By dynamically refining time-domain details, the TS mechanism produces more distinct representations, leveraging the model’s extensive parameter capacity and contextual learning to enhance resilience and adaptability.

#### 3.3.3. Hyperparameter Analysis

**Figure 7:**
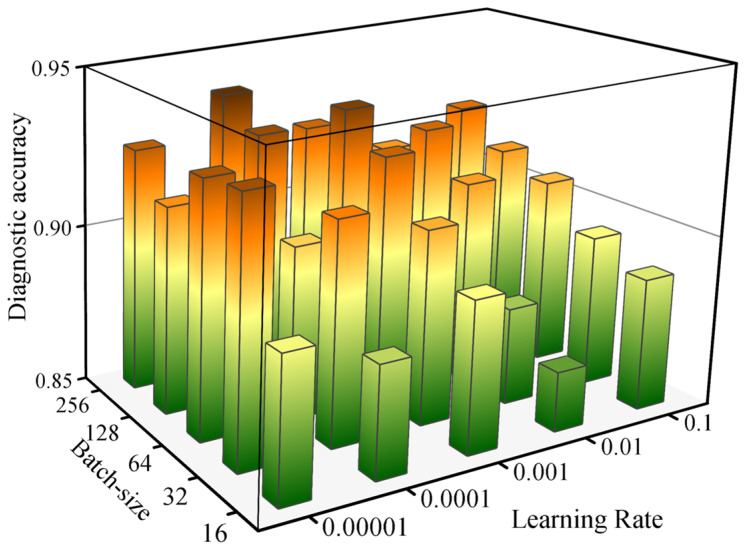
The influence of hyperparameters on the diagnostic accuracy of GLM-4-TS.

From Figure 7, we observe a distinct pattern in GLM-4-TS’s diagnostic accuracy across different batch sizes and learning rates. When the learning rate is relatively high (e.g., 10^−1^), increasing the batch size gradually improves the accuracy, rising from 0.8888 to 0.9209. This suggests that a larger batch size can stabilize parameter updates even under a high learning rate, preventing overly turbulent gradients. However, when the learning rate is moderate (such as 10^−2^ or 10^−3^), although increasing the batch size often leads to a higher accuracy, certain configurations—such as batch size = 256 and learning rate = 10^−2^—show declining performance, implying that “bigger batch” does not always guarantee better results. Notably, at a learning rate of 10^−3^ and batch size = 128, the model achieves an accuracy of 0.9337, indicating a potential sweet spot balancing stable training and efficient gradient updates.

At lower learning rates (10^−4^ and 10^−5^), the situation becomes more nuanced. In general, a larger batch size still tends to yield better accuracy; for instance, with a learning rate of 10^−4^, batch size = 256 reaches 0.9382—over five percentage points higher than batch size = 16. Nonetheless, once the learning rate decreases to 10^−5^, batch size = 32 achieves 0.9295 accuracy, whereas batch size = 128 lags at 0.9137. This outcome can be attributed partly to the slower convergence under lower learning rates: an excessively large batch size may further dilute gradient updates, inhibiting rapid learning. In summary, GLM-4-TS demonstrates strong diagnostic capability in most settings, but optimal hyperparameter choices depend heavily on task requirements, computational resources, and stability considerations. Striking the right balance between the batch size and learning rate is key to achieving both high accuracy and robust convergence.

#### 3.3.4. Cross Verification

**Figure 8:**
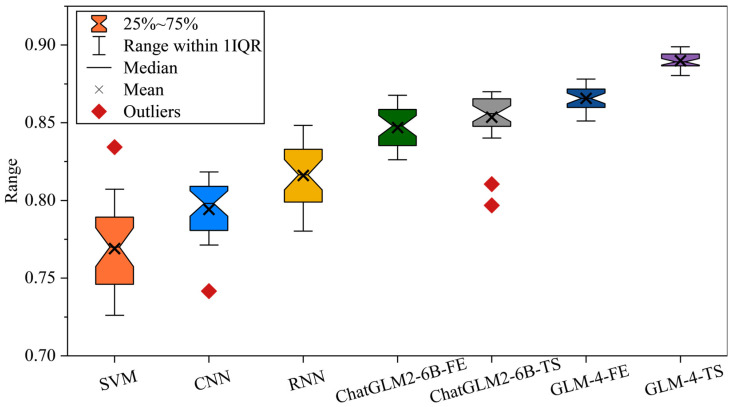
Experimental results based on Case 1 with limited cross-dataset.

From this cross-validation experiment, we observe that traditional approaches (SVM, CNN, RNN), trained on Case 1 and fine-tuned with only 30% of the data from Case 2, generally achieve average accuracies around 0.78–0.84 on the remaining 70% test portion, as shown in Figure 8. While CNN and RNN capitalize on convolutional or recurrent structures to model limited spatiotemporal dependencies and occasionally attain slightly higher performance under simpler fault patterns, their generalization still falls short when faced with complex or noisy operating conditions. In contrast, ChatGLM2-6B-FE and ChatGLM2-6B-TS leverage large-model multi-head attention, with the former accelerating feature aggregation via FE and the latter capturing time-domain bursts through TS fine-tuning. Nonetheless, their accuracies typically hover between 0.83 and 0.86, indicating that discrepancies in task transition and data distribution may constrain their cross-scenario generalization potential. See Table 5.

Within the GLM-4 series, our experiments reveal that GLM-4-FE achieves stable diagnostic accuracies of approximately 0.86–0.88, demonstrating robust feature fusion and cross-domain adaptability. In contrast, GLM-4-TS consistently surpasses accuracies of 0.89–0.90, emerging as the most effective variant. Two principal factors underlie these results: first, the pre-trained model’s rich contextual representation facilitates the discovery of hidden fault patterns across diverse data sources; second, the TS strategy enables flexible adjustment for varying sampling rates and nonstationary signal characteristics, effectively mitigating domain shift. Notably, even with fine-tuning on only 30% of Case 2’s data, GLM-4-TS exhibits strong small-sample transferability and multi-condition synthesis, underscoring its broad applicability in industrial fault diagnosis.

**Figure 9:**
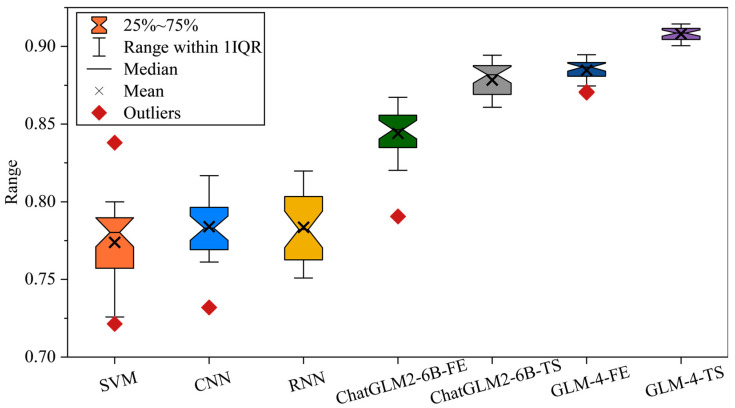
Experimental results based on Case 2 with limited cross-dataset.

In Figure 9, we compared traditional machine learning models, early deep learning models (CNN, RNN), and advanced language models (ChatGLM2-6B-FE, ChatGLM2-6B-TS, GLM-4-FE, GLM-4-TS) in terms of diagnostic accuracy under different data splitting strategies. The experimental setup involved initial training on Case 2, followed by fine-tuning with only 30% of the data from Case 1 and testing on the remaining 70%. The results demonstrate that GLM-4-TS outperforms all other models, achieving an average accuracy of 0.9115, significantly higher than its counterparts. Specifically, the superiority of GLM-4-TS lies in its integration of FE and TS fine-tuning optimization strategies, which enhance its capabilities in feature fusion and temporal information capture. In contrast, traditional models such as SVM, CNN, and RNN, while performing adequately in certain scenarios, generally exhibit lower accuracies and greater variability across diverse and complex fault patterns. The ChatGLM2-6B series models, although improved by incorporating FE and TS, still fall short of GLM-4-TS’s performance, indicating room for enhancement in their feature extraction and temporal adaptation processes.

#### 3.3.5. Training Loss Visualization

**Figure 10:**
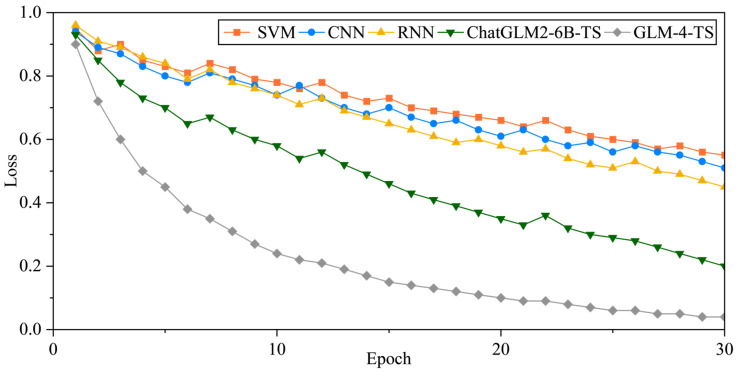
Training losses for each model.

From Figure 10, we can observe distinct convergence patterns for each model. SVM, CNN, and RNN all begin with initial losses around 0.9 and exhibit relatively stable or slow declines in the early epochs. Notably, SVM demonstrates greater fluctuations between Epochs 2 and 3, suggesting that traditional machine learning and early deep learning approaches often require more careful parameter tuning and feature selection when dealing with complex tasks. While CNN and RNN reduce their losses to around 0.70–0.74 by about the 10th epoch, they do not continue dropping quickly thereafter, indicating inherent limitations in their ability to capture nonstationary temporal features and a tendency toward local minima or sensitivity to high-frequency noise. Additionally, CNN experiences a temporary bump at Epochs 7–8, possibly due to instability in the learning rate, kernel configuration, or regularization settings.

In stark contrast, ChatGLM2-6B-TS and GLM-4-TS exhibit significantly faster convergence. ChatGLM2-6B-TS begins with an initial loss of 0.93 in Epoch 1, noticeably lower than the 0.95–0.96 range of the other methods. GLM-4-TS goes even further, starting at 0.90 and dropping its loss to around 0.60 after only three to four epochs—far quicker than CNN and RNN. Such efficiency stems from the capacity of large-scale pretrained models to perform robust feature extraction and precisely characterize time-series bursts, aided by multi-head attention mechanisms. Over subsequent training rounds, GLM-4-TS leverages TS fine-tuning to an even greater extent, driving the loss down to 0.10 by Epoch 20 and eventually stabilizing at approximately 0.04, thus demonstrating superior resilience against noise and dynamic variability. Although ChatGLM2-6B-TS also maintains a steady descent in later epochs, it converges somewhat more slowly than GLM-4-TS. Overall, the results illustrate that advanced large models can achieve fine-grained learning of complex fault patterns in fewer epochs, converging to significantly lower loss levels, thereby underscoring their potent adaptability to high-dimensional, nonlinear time-series data.

**Figure 11:**
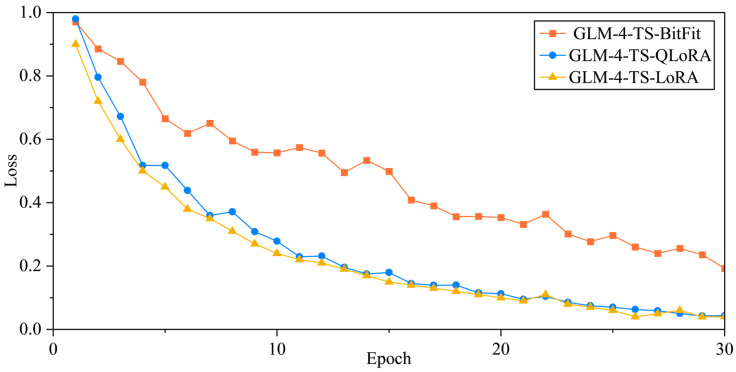
Influence of different fine-tuning methods on training loss.

From Figure 11, we observe distinct convergence behaviors among three parameter-efficient fine-tuning approaches (BitFit, QLoRA, and LoRA) applied to GLM-4-TS. BitFit begins with a loss of 0.9342 at Epoch 1, which is lower than QLoRA’s 0.9757 but slightly higher than LoRA’s 0.90. In subsequent epochs (e.g., Epochs 2–3 and 7–8), BitFit undergoes relatively large fluctuations yet retains a decreasing tendency, reaching 0.5318 by Epoch 10. Compared to QLoRA’s 0.2586 at a similar stage, this is considerably higher, suggesting that BitFit may be less sensitive to certain crucial parameters in a high-dimensional model. Furthermore, BitFit exhibits multiple upward spikes in the later training phase (notably around Epoch 22, rebounding to 0.3837), implying that its long-term convergence may be more susceptible to hyperparameters such as the learning rate and gradient accumulation, ultimately leading to less stability than the other two methods.

In contrast, QLoRA starts at a relatively high loss of 0.9757 during Epoch 1 but rapidly converges in the following epochs, dropping to about 0.4577 by Epoch 5. It continues to steadily reduce its loss beyond Epoch 10, hovering near 0.25 and eventually dipping to approximately 0.0855 by Epoch 23. This reveals QLoRA’s ability to effectively blend fine-grained parameter control with quantization techniques. LoRA, on the other hand, shows a comparatively favorable starting point (0.90 at Epoch 1), swiftly descending to 0.72, 0.60, and 0.50 over Epochs 2–4. Thereafter, it maintains a stable downward trend, attaining 0.24 at Epoch 10 and leveling off near 0.04 by Epoch 30, comparable to QLoRA’s final range. Notably, LoRA’s mid-to-late training exhibits minimal rebounds or oscillations, highlighting its stable adaptation in low-rank updates to multi-head attention and feed-forward layers, enabling the continuous extraction of model potential and suppression of redundant updates. Overall, both QLoRA and LoRA demonstrate robust convergence speed and stability for deep time-series fine-tuning tasks, whereas BitFit may require more rigorous hyperparameter tuning and regularization strategies to unleash its full potential in complex fault diagnosis scenarios.

## 4. Conclusions

In summary, this study introduces a novel end-to-end fault diagnosis framework for milling cutters in intelligent manufacturing by integrating a state-of-the-art large language model (GLM-4) with advanced data preprocessing, feature extraction, and fine-tuning strategies. The framework transforms raw vibration signals into semantically rich representations, thereby bridging the gap between numerical sensor data and linguistic models. The principal contributions and findings of this work are as follows:

**Innovative Semantic Transformation:** The proposed GLM-4-FE module converts complex vibration signal features into interpretable textual descriptions. This semantic transformation enables the model to capture subtle fault characteristics that are often missed by traditional numerical approaches.

**Robust Time-Series Fine-Tuning:** The GLM-4-TS approach leverages multi-head attention and temporal adaptation to extract both local and long-range dependencies from time-series data. The experimental results on both the PHM 2010 dataset and our proprietary milling cutter dataset demonstrate diagnostic accuracies exceeding 93%, even under high-noise conditions (SNR as low as –10 dB) and with limited training samples.

**Comprehensive Comparative Analysis:** Compared with traditional methods (e.g., SVM, CNN, and RNN) and baseline large language models, our framework consistently outperforms in terms of accuracy, robustness, and generalization. This improvement underscores the advantage of integrating domain-specific feature engineering with the semantic reasoning capabilities of large language models.

Despite these promising results, certain limitations remain. The current framework has been validated primarily in single-tool scenarios; therefore, extending the approach to handle multi-tool systems and cross-scenario generalization remains a challenge. Additionally, while the fine-tuning process is efficient, the further optimization of hyperparameters and exploration of data augmentation techniques could enhance performance under even more challenging conditions. Future research will focus on the following:

**Multi-Tool System Extension:** Expanding the framework to accommodate multiple tool types and varying operational conditions, potentially through federated learning strategies to support distributed industrial deployments.

**Advanced Data Augmentation:** Investigating the integration of generative adversarial networks (GANs) and other augmentation methods to further address the issues of small sample sizes and extreme noise environments.

**Hybrid Model Development:** Exploring models that combine both feature-based and raw time-series inputs to fully exploit complementary information and further boost diagnostic accuracy.

In conclusion, the proposed GLM-4-based fault diagnosis framework provides a scalable, interpretable, and high-performance solution for real-time tool health monitoring. By bridging the gap between the theoretical advancements in large language models and practical manufacturing demands, this work lays a strong foundation for next-generation intelligent fault diagnosis systems in industrial applications.

## Figures and Tables

**Table 1:** Feature extraction formula and description.

Feature Domain	Feature Name	Mathematical Expression	Physical Meaning
Time Domain	Mean Value (MV)	$X_{1}=\frac{1}{N}\sum_{i=1}^{N}\left|x_{i}\right|$	The average trend of signal amplitude variation.
	Root Mean Square (RMS)	$X_{2}=\sqrt{\frac{1}{N}\sum_{i=1}^{N}{x_{i}}^{2}}$	The mean energy of the signal over a given time interval.
	Standard Deviation (SD)	$X_{3}=\sqrt{\frac{1}{N}\sum_{i=1}^{N}{\left(\left|x_{i}\right|-X_{1}\right)}^{2}}$	The degree of fluctuation of the signal around the mean.
	Skewness Factor (SF)	$X_{4}=X_{2}/X_{1}$	Variations in the signal waveform.
	Skewness (Ske)	$X_{5}=\frac{1}{N}\sum_{i=1}^{N}{\left(\frac{\left|x_{i}\right|-X_{1}}{X_{3}}\right)}^{3}$	The degree to which the signal distribution deviates from the mean symmetry line.
	Kurtosis (Kur)	$X_{6}=\frac{1}{N}\sum_{i=1}^{N}{\left(\frac{\left|x_{i}\right|-X_{1}}{X_{3}}\right)}^{4}$	The smoothness of the signal waveform.
Time Domain	Peak Value (PV)	$X_{7}=max\left|x_{i}\right|$	The maximum instantaneous amplitude of the signal.
	Crest Factor (CF)	$X_{8}=X_{7}/X_{2}$	The extremity of the peak in the signal waveform.
	Impact Factor (IF)	$X_{9}=X_{7}/X_{1}$	The instantaneous impact characteristics of the signal.
Frequency Domain	Mean Power Spectrum (MPS)	$X_{10}=\frac{1}{N}\sum_{i=1}^{N}P\left(f_{i}\right)$	The variation of signal power with frequency.
	Frequency Center (FC)	$X_{11}=\frac{\sum_{i=1}^{N}f_{i}\times P\left(f_{i}\right)}{\sum_{i=1}^{N}P\left(f_{i}\right)}$	The static portion of the spectrum.
	Mean Square Frequency (MSF)	$X_{12}=\frac{\sum_{i=1}^{N}{f_{i}}^{2}\times P\left(f_{i}\right)}{\sum_{i=1}^{N}P\left(f_{i}\right)}$	The degree of fluctuation of the spectrum near the frequency centroid.
Time–Frequency Domain	Wavelet Packet Energy (WPE)	$X_{13}=\frac{1}{N}\sum_{i=1}^{N}{\left[d_{i,k}^{\left(M\right)}\left(t\right)\right]}^{2}$	The average energy of the signal at different scales.

**Table 2:** PHM 2010 competition experiment parameters.

Parameter	Category	Parameter	Category
Model	Roders Tech RFM 760	Radial cutting depth	0.125 mm
Workpiece material	Nickel-based superalloy 718	Axial cutting depth	0.2 mm
Cutter/Tool	3-Tooth ball nose milling cutter	Number of sensors	3
Spindle speed	10,400 RPM	Sensing channels	7
Feed rate	1555 mm/min	Sampling frequency	50 HZ
Cutting speed	5000–20,000 rpm	Tool diameter	6–12 mm

**Table 3:** Average diagnostic accuracy of each model in Case 1.

Model	Diagnostic Accuracy
SVM	$0.832_{-0.275}^{+0.325}$
CNN	$0.858_{-0.098}^{+0.157}$
RNN	$0.854_{-0.195}^{+0.267}$
ChatGLM2-6B-FE	$0.883_{-0.116}^{+0.215}$
ChatGLM2-6B-TS	$0.884_{-0.076}^{+0.135}$
GLM-4-FE	$0.898_{-0.048}^{+0.093}$
GLM-4-TS	$0.918_{-0.052}^{+0.069}$

**Table 4:** Average diagnostic accuracy of each model in Case 2.

Model	Diagnostic Accuracy
SVM	$0.853_{-0.295}^{+0.382}$
CNN	$0.864_{-0.265}^{+0.329}$
RNN	$0.871_{-0.235}^{+0.275}$
ChatGLM2-6B-FE	$0.904_{-0.235}^{+0.215}$
ChatGLM2-6B-TS	$0.915_{-0.153}^{+0.175}$
GLM-4-FE	$0.922_{-0.116}^{+0.135}$
GLM-4-TS	$0.938_{-0.061}^{+0.079}$

**Table 5:** Division of training and test dataset.

No.	Train	Transfer Dataset	Test Dataset
1	Case 1	30% Case 2	70% Case 2
2	Case 2	30% Case 1	70% Case 2

## Data Availability

PHM 2010 is available at https://phmsociety.org/phm_competition/2010-phm-society-conference-data-challenge (accessed on 30 March 2025).

## Abbreviations

The following abbreviations are used in this manuscript:

Abbreviation	Definition
CNC	Computerized Numerical Control
CNN	Convolutional Neural Network
ChatGLM2	Chat GLM Version 2
LLM	Large Language Model
GLM-4	General Language Model 4
RNN	Recurrent Neural Network
SVM	Support Vector Machine
SNR	Signal-to-Noise Ratio
LoRA	Low-Rank Adaptation
QLoRA	Quantized LoRA
PHM	Prognostics Health Management
